# Engaging in Action Research with Nurses: Overcoming Challenges and Gaining Positive Insights into End-of-Life Care

**DOI:** 10.3390/nursrep14030115

**Published:** 2024-06-21

**Authors:** Yuka Oura, Shiori Kato, Risa Kaino, Yasuna Sato, Junko Shida, Chisaki Uno, Yumi Matsuda

**Affiliations:** 1 Long-Term Care Unit, Yamagata Tokushukai Hospital, 2-3-51, Kiyozumicho, Yamagata 990-0834, Japan; 2 School of Nursing, Yamagata University, 2-2-2 Iida-Nishi, Yamagata 990-9585, Japan; c.uno@med.id.yamagata-u.ac.jp (C.U.); yumi-m@med.id.yamagata-u.ac.jp (Y.M.)

**Keywords:** action research, case research method, evidence-based practice, positioning

## Abstract

The aim of this study was to qualitatively describe, from a practitioner’s perspective, the process by which nurses struggle to support a patient with end-of-life cancer with frequent nurse calls and gain positive insights through two methodologies: AR and the case study method. The participants were four ward nurses who supported a patient receiving end-of-life cancer in his 80s. The participants engaged in monthly group work and practical training sessions, which included facilitators, to reflect on and develop care plans. Based on these activities, care was provided to the patient. After the intervention period, the patient’s course and practice was documented and analysed qualitatively. The intervention significantly improved the nurses’ ability to support inpatients with many needs through careful observation, enhancement, and practical skill improvement. This process resulted in a better understanding of patient needs, proactive skill development, enhanced team performance, and an innovative care-delivery system that resonated throughout the ward. This study demonstrated a successful strategy for nurses to improve support for high-need inpatients, emphasising the importance of attentive care, proactive skill improvement, and a team-based approach to healthcare innovation.

## 1. Introduction

Nurse calls are a tool and means for patients to communicate their intentions and requests to staff at remote locations in healthcare facilities. Although nurse calls are an essential patient lifeline, frequent nurse calls can confuse staff. For example, nurses are confused, sometimes frustrated, and even angered by incessant calls and detailed and varied complaints from patients with amyotrophic lateral sclerosis [1]. The impetus for this study also came from a stalemate in which nurses and carers working in long-term care wards were ‘exhausted by the frequent calls from a certain patient, day and night, and did not know how to respond to them’.

In this study, the ward nurses experienced similar challenges. They were often exhausted by frequent calls from a certain patient around the clock and were uncertain how to respond. This led them to seek advice from researchers specialising in nursing science, which ultimately served as an impetus for the commencement of this research.

Given the challenges faced by ward nurses in addressing patient care issues and the necessity for problem solving, we initiated a collaborative effort using the action research (AR) methodology. AR is a research design method that focuses on specific events in specific settings to explore problems and seek solutions in collaboration with the individuals involved in those settings [2]. A characteristic feature of AR is its integrative approach to practice and research, in which insights gained from practice are immediately applied, evaluated, and iteratively improved [2,3]. AR has been applied to various fields, including nursing, education, social welfare, and organisational reform, aimed at causing sustainable change through interactions between practice and theory [3].

In nursing, including elderly and end-of-life care, AR has been extensively utilised by care providers, yielding numerous insights. For instance, AR has been successfully applied in long-term care facilities, where advanced care planning (ACP) is minimally practiced. In these settings, AR facilitates the development of a resident- and family-centred ACP process model that respects residents’ religious beliefs [4]. Additionally, AR has supported staff in long-term care facilities struggling with end-of-life care for patients with dementia by guiding pain assessment and management, medication management, and nutritional and hydration care [5].

These examples demonstrate the efficacy of using AR to introduce new systems and support staff facing challenges in providing diverse and individualised care. In this study, it was necessary to identify and address the underlying issues faced by nurses confused by frequent calls from a terminally ill patient. Considering the strengths of AR that have been demonstrated in previous research, including its effectiveness in supporting staff to meet patient needs, AR was deemed an appropriate method for this study.

However, challenges associated with AR have also been highlighted. A significant issue pertains to data collection and analysis. Specifically, when handling subjective data, ensuring the reliability and validity of data collected throughout the AR process has been identified as a challenge [6]. Another challenge is the sustainability of implemented changes. Without the continuous support and commitment of stakeholders, improvements are often temporary. Thus, it is crucial to create an environment in which participants’ contributions are recognised, changes are integrated into daily practice, and continuous evaluation is maintained [6].

Considering these challenges, this study emphasises the need to align core AR processes—reflection, planning, and implementation—with reliability and validity. Additionally, it is necessary to foster an organisational culture that supports the sustainability of change by encouraging participants to support each other even after the AR process has been concluded. The approach we focused on was a case study research method for developing knowledge of the expert care practice method (the case study method) advocated for by Yamamoto [7].

This case study employs a new method of case study research developed in Japan in 2018 that utilises field discoveries to create practical content that can be shared with others while ensuring academic rigor. By making practitioners aware of the importance of nursing and verbalising the value of their practice, the article’s readers can relive the practice, and the significance of shared behavioural changes, experiences, and worldviews is demonstrated [7].

Although this research method is relatively new, and prior studies are sparse, some notable examples exist. One study examined the process of home-based end-of-life care for patients with intractable pressure ulcers, detailing family support provided by visiting nurses from a practitioner’s perspective [8]. Another study reviewed discharge support that led to home transitions for patients and families unprepared for home care. It revealed nurses’ intentions and the meaning of their assistance, which were not consciously recognised during practice [9]. These studies highlighted the potential of this method for providing practical nursing knowledge.

In previously published studies, reflection typically began only after the completion of care. However, this study undertakes a novel approach by initiating an analysis using this case study method, not only after the completion of practice but also during the AR process. This approach is expected to help nurses recognise the meaning and value of their practice throughout the AR process, allowing them to apply these insights directly to patient care. Additionally, this method aims to overcome the challenge of ensuring the reliability and validity of subjective data in AR [6], thereby elucidating the practical knowledge involved in overcoming the challenges nurses face.

The rationale for this approach is that the practical knowledge articulated through the case study method is centred on care providers [7]. It is presumed that nurses gain a sense of accomplishment from and find meaning in their care practices by verbalising their experiences while overcoming challenges. Previous research has indicated that when nurses gain a sense of accomplishment and find meaning in their support activities, they build confidence and a sense of achievement, equipping them with resilience to face challenging nursing situations again [10,11].

Given these considerations, AR and the case study method are anticipated to enable nurses to perceive a tangible sense of the responses, meaningful reactions, and outcomes resulting from their actions and approaches. This realization is expected to foster positive emotions, motivating nurses to enjoy their practice and continuously strive to provide better care [12,13]. Additionally, the ripple effect of this motivation could further enhance the quality of care provided.

Moreover, the combination of AR and the case study method is a novel approach in nursing research, as no existing studies have concurrently utilised both methodologies. Thus, this study has the potential to provide valuable insights as a practice-based research initiative to advance the field of nursing science.

This study aimed to qualitatively describe, from the practitioner’s perspective, the process by which nurses struggled to support a patient with terminal cancer who made frequent nurse calls and gain positive insights through two methodologies: AR and the case study method.

## 2. Materials and Methods

### 2.1. Overview of Participating Institution

The study site was a medical facility in Prefecture A in the Tohoku region, and the ward where the AR was conducted was a medical care bed. In addition to nurses, nursing staff and others assist daily.

### 2.2. Design

**Figure 1:**
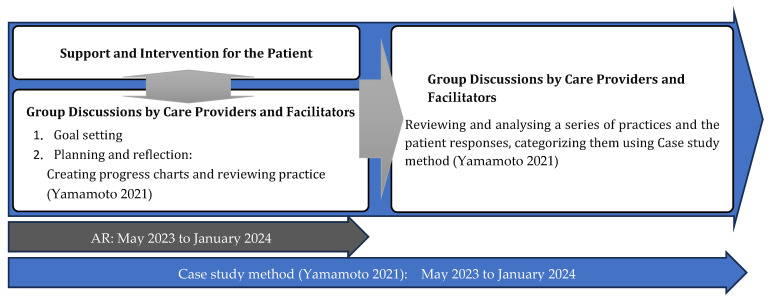
The process of this research. Reference: [7].

This research design integrates prospective and retrospective approaches by leveraging two methodologies: action research (AR) and a case study (Figure 1).

### 2.3. Participants

The study involved four nurses (with an average of 12.8 years of experience) who supported Patient A as part of the AR process and participated in discussions using the case study method after the completion of AR. Nurses who supported Patient A but did not meet these conditions were excluded from the study and are referred to as staff. The practical team included three university lecturers specialising in nursing.

### 2.4. Case Summary

The patient is a man in his 80s. He had chronic renal failure and multiple metastases from colorectal cancer and was in the end-of-life stage. He was hospitalised due to a cervical spine injury caused by a fall down the stairs at home, which, combined with his declining cognitive function, led to him being bedridden. In addition to the physical and psychological distress caused by the terminal phase, as his hospitalisation progressed, he gradually developed contractures in his limbs, making it difficult for him to move.

### 2.5. Methods of Data Collection and Analysis

The intervention period extended from May 2023 to January 2024, and the intervention period for the patient extended from May to October.

The AR research team consisted of four nurses and three facilitators who were university lecturers specialising in nursing. The participants were members of staff responsible for nursing research in the institution, and the institution asked one facilitator to participate as a nursing research facilitator.

During the intervention period, from May 2023 to October 2024, group work, including facilitator work, was usually conducted once a month, four times. Based on the facilitator’s guide [2], the facilitator asked and elicited narratives from the participants with the intention of (1) encouraging narratives, (2) shedding light on the practices, and asking them to tell us why and how they did so, and (3) putting the meaning and intentions of the practices into words that would convey them to the reader. Additionally, one participant attended six training sessions led by the facilitators and others.

#### 2.5.1. Setting Action Research Goals

From the initial group work, the goal of the AR was set as ‘to gain positive insights through supporting the patient with frequent nurse calls, that is, to capture the patient’s reaction to the nurses’ actions and approach and to get a good feeling for the practices’.

#### 2.5.2. Group Work during Action Research (Reflection and Care Planning)

In the group work, the practice content at the time of the group work was reviewed using nursing records, among others, and a progress chart was made. The progress chart consisted of the patient’s situation, what the participants thought, the content of the practice, the intention and implication of the practice, and the patient’s reaction. It was written in chronological order. In the group work, the participants discussed questions about the diagram’s content, and comments made during the discussion were added to the worksheet. Additionally, the participants discussed the patient’s reaction and the event’s meaning, including the thoughts and intentions that led to the decision at that time. The content of these discussions was also written on the worksheet. This corresponded to reflection on practice; on this basis, a care plan was drawn up with the participants’ agreement.

#### 2.5.3. Group Work after the Intervention Period (Categorisation of Practices)

After the end of the intervention period, based on the progress chart, the patient’s progress and practice development were described in as much detail as possible using a case study worksheet. The descriptions were transcribed and coded so that the semantic content was not compromised, and they were grouped with regard to similarities and differences. Subcategories were then formed and named, clearly indicating practices in terms of their intentions. Furthermore, each period separated in the case study method was called a turning point, and a table was created with the period on the horizontal axis and the category of practice on the vertical axis. This was conducted using the case study method [2]. The table was discussed among the members, and the final version was one in which all members agreed on the relationships between the subcategories, categories, and practices.

### 2.6. Ethical Considerations

The Ethical Review Committee of the hospital to which the researcher belongs approved this study (approval number 2023-5). The purpose, research methods, voluntary participation, confidentiality issues, and plans for publishing the research results were explained to all participants in writing and orally. Written informed consent was obtained from all participants.

For Patient A, the aforementioned ethical considerations were explained both orally and in writing, and consent was obtained through the signing of the consent form.

## 3. Results

The analysis revealed how practitioners gained positive insights and practical knowledge. Below are the categories, with [ ] and <> indicating subcategories, typical practice descriptions (codes) in ‘ ’, and facilitator comments in “ ”.

### 3.1. Process of Gaining Positive Insights through the Action Research (Figure 2)

The process of gaining positive insights through AR is divided into four stages. Stage I is when participants pay attention to the patient and carefully observe the patient’s speech, behaviour, and facial expressions, among other aspects, to grasp what the patient is experiencing accurately. When the participants realised that the positioning they were performing daily did not alleviate the distress caused by the patient’s lack of positioning, they realised that the positioning techniques that were being used to alleviate distress in Stage II were inadequate and that there was a limit to how far this inadequacy could be resolved in the ward. Therefore, it was time for them to take proactive steps to improve their ability to provide care that would relieve their patient’s distress by attending training sessions. However, the patient’s suffering was not alleviated at this point, and the staff experienced confusion and conflict.

**Figure 2:**
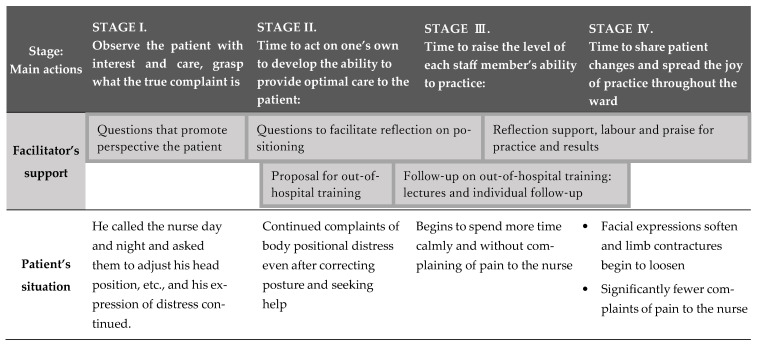
The process of action research.

A turning point occurred in Stage III. Based on the knowledge gained from external training in Stage II, the participants considered improving each staff member’s ability to care for the patient. Individual training was conducted again in the ward, and the participants realised the effectiveness of the training. This careful individual follow-up brought about changes in the care of each staff member. In the final Stage IV, when the patient’s recovery through care was captured, the entire ward became more motivated to enjoy the practice and provide daily support. Thus, AR’s goal was achieved.

### 3.2. Practical Knowledge Gained by Practitioners Overcoming Difficulties and Gaining Positive Insights (Figure 2, Table 1, and Supplementary Materials)

Four categories and twelve subcategories were extracted across the four stages.

**Table 1:** Categories of the practices.

Categories	Subcategories	Example of a Code
(1) Feeling and thinking about suffering where the subject is the patient.	(1) Why call the Nurse? Look at the reasons why.	We were so bewildered by the number of nurse calls that we did not look at the background behind the patient’s pressing the nurse call. (STAGE I)
	(2) Observe the patient thoroughly and assess the situation, with or without a nurse call	Regardless of whether the patient called for a nurse, we gathered information about the content of his complaints and facial expressions and dealt with them. (STAGE I)
	(3) Knowing how hard it is for the patient to position himself.	The complaints were mostly about position. I could tell he was in a lot of pain. (STAGE I)
	(4) Accept the fact that the care was not relieving the patient’s pain	The number of nurse calls did not change even after adjusting the position, and this did not reduce the patient’s distress. (STAGE I)
(2) Seek care tips from ourselves to improve our practice	(5) Request training to PTs in the hospital to overcome the problem of positioning	Let us ask the PT so that I can position myself in a way that would suit the patient. (STAGE II)
	(6) Encountering care that I want to practice! I can do this	I learned at the no-lifting training that the care I thought was good for me was actually bad for me. I thought I was doing it all the time. (STAGE II)
(3) Spend time on training that touches your heart	(7) Consider training methods that would move the hearts.	Training and handing out materials that convey knowledge in a light-hearted manner will not generate interest or need. (STAGE III)
	(8) Steady individual follow-up until everyone realizes the difference and effectiveness of care.	If everyone feels the difference and comfort of care, it will lead to better care, and if that is the case, one-on- one practical exercises are necessary. (STAGE III)
	(9) I am impressed with the comfort of the positioning! Share the feeling of “I want to give this care!”	Everyone is surprised and impressed by the difference in positioning. The atmosphere in the wards has changed as training has progress. (STAGE III)
(4) Create an organization that can provide new positioning care as a team	(10) Everyone can confidently provide at least one form of care based on evidence.	Not somehow but think about putting a pillow here so that you can rest your body safely, so that you can do it with a rationale. (STAGE IV)
	(11) Confirm the essence of the ‘must-do’ mindset and techniques and calling out to each other	‘Focusing on the basics, such as supporting pillows with a stable surface rather than supporting them, so that everyone can put them into practice. (STAGE IV)
	(12) Capture the pleasant changes that occurred in the patient and share them with all the staff	Everyone says the contractions have loosened and blood pressure readings are easier. (STAGE IV)

(1) [Feeling and thinking about suffering where the subject is the patient].

This was a common category extracted through all stages and described the nurses’ attitudes towards relieving the patient’s distress by paying attention to the patient and observing the patient’s words, actions, and facial expressions.

Participants who responded to the patients’ frequent nurse calls, day and night, were confused and sometimes frustrated that they were ‘being called again’ and that they had ‘other patients to take care of’. In response, the facilitator thanked the participants for their efforts and asked, “Why does the patient push the nurse call so hard?”. One participant thought silently for a moment and then reflected ‘there were a lot of complaints about the position of the head’, which ‘are primarily about positioning, such as repositioning’. Their perspective began to shift towards the patient. The participants then realised that ‘the extent of his suffering, which he could not manage on his own, was manifested in the number of nurses calls he received’. Furthermore, the facilitator asked, “What does the patient look like when not pressing the nurse call?” ‘Sleeping peacefully?’ The participant said, ‘in contrast (no nurse call), I may not see him enough at those times, and I do not have much time to spare’.

Through these reflections, the participants realised that the patient’s frequent nurse calls, which had been expressed as a problem for them, had somehow become a problem for the staff. This led to a change in their mindset to focus on the patient’s real problems and the need to determine why he could not resist pressing the nurse’s call button. These are the reasons for the practice of <paying attention to why he presses the nurse call>, which led to the development of a plan to <observe the patient thoroughly and assess the situation, with or without a nurse call>. In practice, the staff paid attention to the patient’s living conditions not only when he directly complained about something but also during rounds, mealtimes, and at any other time possible and continued to provide positioning support as a practice to alleviate his pain.

Consequently, the participants gained a new appreciation for how difficult it was to position the patient. Most of his complaints were related to positioning, and the participants, being aware of the patient’s painful situation, provided support, such as changing the position of his head at his request each time. However, even with positioning support, pain relief did not continue, and the patient continued to complain of pain resulting in frequent nurse calls. These realities forced the staff to ‘accept that care did not relieve the patient’s pain’.

(2) [Seek care tips from ourselves to improve our practice].

This category indicates a proactive attitude towards actively participating in training programs within and outside the hospital to increase the level of knowledge and skills that are lacking, as they feel that there is a limit to the provision of positioning that reduces the patient’s pain by the ward staff alone, including the participants. In Stage I, the participants reflected on the content and method of care concerning the patient’s inability to relieve his positional distress, saying that ‘if he calls the nurse immediately, something must be missing or wrong with our care’. The participant then said that ‘we can no longer solve the problem ourselves; if it is positioning, we will ask a physiotherapist (PT)’, coming up with the idea of taking positive action to learn about other methods.

In response, a plan was drawn up to <request training to PTs in the hospital to overcome the positioning problem>. The staff, including several participants, received training in using positioning pillows directly from the PT. Posting pictures of positioning examples at the bedside to standardise care ‘ensured that staff who could not participate in the trial could also practise’. However, there were some puzzling comments, such as ‘sometimes I say it is good, but it does not last long’, and ‘if anything, it does not change much... I do not know what’s wrong with the care’. The participants’ expressions were unchanged, and there was a heavy atmosphere during the group work. However, there was a feeling that ‘we could not stop there; we had to keep going’, and the group decided to continue exploring the reasons for not being able to alleviate the patient’s distress.

At this point, the facilitator conducted a no-lift training workshop that was took place outside the hospital. This training was led by members of the university faculty, including the facilitator, and was held approximately once or twice a month for six sessions. In Stage II, one participant proactively attended an out-of-hospital no-lift training session. However, Stage II consisted mainly of classroom lectures on the principles of positioning, which did not change the patient’s positioning support.

(3) [Spend time on training that touches your heart].

This category relates to the aspect of taking action to deliver training, which is not just a knowledge transfer type of training but one in which each individual understands the comfort that can be gained from positioning and is moved to want to practice and be able to provide that care.

During the out-of-hospital no-lift training, the participant began attending Stage II and practical skills training. In the practical skills training, she reproduced the posture of a patient who was experiencing difficulty with positioning and implemented a new practice using positioning pillows and pressure relief before and after positioning based on the basics she learned. She mimicked the patient’s contractures and positioning and shared their concerns and specific problems with the facilitator and other training participants. She was shocked to learn that the care she thought was good was unsatisfactory. Simultaneously, she was ‘moved by the comfort of the positioning, and her heart was moved to try this care on the patient as soon as possible tomorrow’.

In addition to this experience, she reflected on the factors that prevented them from using PT training to provide adequate support. Consequently, she said, ‘The basics are essential, but I was looking for an easy way to do it’, and ‘the PT taught me the basics. However, we neglected to understand the basics, and there was a lack of follow-up for staff who could not attend the training’. In particular, she attended two training sessions inside and outside the hospital. She realised that ‘even though the pillow position and other aspects of the pillow taught by the PTs looked the same, they were not able to use the pillow to support the weight of the body and distribute pressure’ and that ‘sometimes just copying the look or type does not lead to correct practice. However, once people experience this comfort, they will all want to put it into practice’. She clearly gained intuition and conviction.

Her experiences were shared in the group work to <consider training methods that would move the hearts>. Therefore, in addition to ‘positioning the patient, which has been practised thus far, even if it takes time’, a training plan was developed to ‘enable each staff member to experience comfortable positioning based on principles’ and to <steady individual follow-up until everyone can feel the difference in care and its effects>.

All the staff who took part in the training were shocked, as were the participants, to find that ‘the positioning they had practised was completely different from the new positioning’. They also experienced first-hand the pain that the patient had been experiencing, which led to a fundamental understanding of how he felt compelled to press the nurse’s call button, saying that this ‘(this position) is too tight, I cannot stay in this position, even for a short time...’. These experiences increased the staff’s motivation to ‘want to provide this care and bring comfort to the patient through this care’. As the workshop progressed, all staff members were impressed by the comfort brought by the positioning. They all wanted to provide such care. As such, the atmosphere in the ward improved.

(4) [Create an organisation that can provide the new positioning care as a team].

This category represents the creation of an organisational environment in which the newly introduced positioning care is not a unique skill possessed by a few providers but can be acquired and maintained by all staff in the ward as an organisation after Stage III.

The plan is based on a shared understanding of critical principles that must be maintained and implemented. The plan has been formulated to <confirm the essence of the ‘must-do’ mindset and techniques and call out to each other>. Each member of staff who learned the principles of positioning and experienced the comfort of positioning through the individual follow-up said, ‘I do not just look at a picture and think ‘the pillow is here’, but I do it with a basis in mind, such as ‘the pillow should be firmly supported where this pressure is applied’. At first, it took time to think about it, but after repeatedly repeating the process’, <everyone can confidently provide at least one form of care based on evidence>.

As a result of these efforts, which spread throughout the ward, the patient’s expression, which often left him with a distressed look on his face even after repositioning, eased, and staff began to see the impact of the care they were providing: ‘The first request to reposition his head has disappeared’. Nurse calls about body positioning genuinely decreased. The staff felt the impact of the care provided. Moreover, ‘the patient’s contractures loosened a bit, and measuring blood pressure in his upper limbs became easier’. The staff felt the physical changes caused by the positioning. Thus, the staff could <capture the pleasant changes in the patient and share them with all the staff>. The group work in Stage IV did not include the unpleasant expressions and heavy atmosphere that prevailed up to Stage II, and the practitioners always smiled. The facilitator added only a few questions at this Stage to explore the intention to care during reflection, and the focus was on sharing experiences and providing praise for practice and results.

## 4. Discussion

In this study, participants struggling to provide end-of-life care to a patient making frequent nurse calls began to gain positive insights through AR and the case study method by working with the facilitator. The four stages of the process were (1) paying attention to the patient and observing him to understand his real needs, (2) taking the initiative to improve technical skills, (3) improving the practical skills of each staff member, and (4) sharing changes with the patient and spreading the joy of practice throughout the ward.

Additionally, the following categories were presented as practical knowledge to understand the response: (1) grasping suffering with the patient as the subject, (2) actively acting on one’s own to improve practical skills, (3) making the training resonate with each staff member, and (4) creating an organisation that can provide new care as a team. Through these processes, the participants moved out of the deadlock and experienced confidence in the care they provided, a sense of accomplishment, and a successful experience. The processes corresponding to acquiring skills based on the principles of positioning and their application to the patient showed aspects of developing evidence-based practice (EBP) as an organisation.

The participants in this study were nurses who struggled to respond to a patient who frequently complained. In responding, as in previous studies, the practitioners were confused, sometimes frustrated, and angry [1]. Such situations are typical in clinical practice. However, without escaping the impasse, the participants honestly confronted their skill deficits, including the patient’s, learned new care skills through staff collaboration, and accumulated successful experiences. Thus, this study was significant in that it supported practitioners and (1) visualised the process by which practitioners facing difficulties in supporting the patient were able to gain positive insights on their own, and (2) described the process of practising EBP by identifying patient needs and effectively disseminating new care skills needed to meet those needs throughout the organisation. The significance of this report lies in the fact that it describes the process of practising EBP by identifying patient needs and effectively disseminating the new care techniques needed to meet those needs throughout the organisation.

### 4.1. Effect of Using Both Action Research and the Case Study

This study uses AR [2] and the case study method [7]. AR is a prospective study technique [2] in which practitioners and researchers work together to solve the current problem. Conversely, the case study method [7] is a retrospective study that considers the support already provided. However, this study is unique in that it involves group work in which the participants carefully reflected on their practice after completing a set of supports and throughout the AR process. This facilitated deeper ongoing reflection, planning based on that reflection, and practical training. Furthermore, it was believed to offer deeper insights through continued team reflection after the practice concluded. Yamamoto [7], the developer of the case study method, also found it compelling to incorporate a part of the case study method into conferences and other settings, suggesting that integrating the case study method into the practice process is effective.

Thus, in this study, group discussions led to a more effective practice. The facilitator played a vital role in this discussion. The facilitator participated in the group work by not denying the practice’s content or the participants’ feelings based on the facilitator’s guide [7], respecting the practice, and trying to create an environment in which it was easy to talk. It was thought that these cumulative efforts led to the participants feeling that ‘it is safe to speak one’s true feelings here’ and facilitated the building of an open team [14], which is the basis of the practice.

Additionally, the facilitator in this study was an external facilitator with a different affiliation to the participants, characterised by being a certain distance from the site and acting as a facilitator with a bird’s eye view. It was thought that the realisation that collaboration can occur when the participants have good relationships, allowing them to capture the details provided here, brought stability to the individual members and the team and acted as a driving force [14] for them to transform the practice as a team. Therefore, once in Stage IV, the facilitator mainly provided support and praise for the practice and results. 

Culturally, Japanese people tend to be modest and underestimate themselves, and self-deprecation is likely to occur [15]. Therefore, others need to provide legitimate recognition and feedback on their achievements, which is an essential source of support. In this study, the support of an external facilitator specialising in nursing likely encouraged participants to have confidence and pride in the process of providing and the outcomes of their care. This set of processes contributes to achieving AR goals and EBP-based practices. Moreover, by the end of the process, the team had developed into one that could practice EBP to meet patient needs autonomously, without facilitator support, in response to the goals of the AR. The maturity of the members and team can be assessed by a change in the role of the facilitator, which is another outcome that supports the achievement of AR goals.

This study examines aspects of building EBPs and developing their functions as a team. It should not be forgotten that these were made possible by the collaboration between practitioners and facilitators, including the support system of the organisation in question. Organisational support is essential [14], along with facilitators who provide stimulation and inspiration for the construction of ARs and EBPs, such as those in this study. The beliefs and actions of the nurse managers, who considered the presence of highly knowledgeable and skilled facilitators essential for practice and research, formed the basis for the findings of this study.

### 4.2. Practical Knowledge for Introducing New Technologies throughout the Organisation

The participants in this study faced two challenges: the first was an obvious challenge in responding to frequent nurse calls, and the second was a potential challenge in acquiring new care technologies and disseminating them throughout the organisation. Although the two issues appear to be different, we believe that the reason for solving both problems is that they are intertwined within several processes. In particular, in the case of the first issue, ‘difficulty in responding to frequent nurse calls, participants understood the pain of the patient who could not help pressing the nurse call button and reflected on the fact that the current knowledge held by the entirety of the staff could not solve this pain. Simultaneously, the participants believed that ‘we want to alleviate suffering somehow’ and that ‘we are the ones who can provide care to alleviate suffering’. These factors were seen as the driving forces to change the current situation and increase the momentum to spread the adoption of new care techniques across wards.

With the introduction of new care techniques, staff resistance increased. This is because it is easy to generate negative feelings and scepticism [16], such as ‘What is wrong with the existing technology’, ‘It takes time and effort to learn new things’, and ‘Are the benefits truly worth the effort?’ Therefore, resistance to various types of change within organisations is seen as an inevitable phenomenon, and overcoming resistance to change is essential for successful change and smooth organisational reform [16]. In this study, the introduction of new care technology—that is, the positioning of support that is different from what has been done in the past—corresponds to transformation. For practitioners, some staff members were expected to be reluctant to acquire a new assistive technology because it would negate the positioning support they had previously provided. In this case, skills acquisition would be limited to several staff members, meaning that high-quality support methods would not be disseminated throughout the organisation. In the long term, this problem is detrimental to patients.

However, in this study, there was no significant resistance or conflicting opinions from the staff, and, especially after the individual follow-up training in Stage III, the acquisition of skills proceeded smoothly. The reason for this was thought to be that staff throughout the ward experienced the patient’s pain and were able to share their understanding that the current techniques were not sufficient to relieve his pain and that there were effective techniques that could replace them through participation in the training sessions, and that they could acquire adequate skills. These factors were seen as the driving forces that enabled the whole organisation to learn positioning techniques and support patients with a sense of speed. Furthermore, the patient was at the end-of-life stage of his cancer, and the staff’s sense of urgency that their remaining time was minimal may have increased their motivation to learn the technique.

Additionally, interprofessional work (IPW) and care were organised. In this case, discussions between the participants were emphasised from the outset, and they continued to be involved, mutually validating each other’s facts, feelings, and thoughts. For example, all the staff shared and empathised with the patient’s initial difficulties and exhibited exhaustion in responding to nurse calls without denying their feelings of frustration. Therefore, group work, in this case, can be seen as a process that promotes understanding and respect for others in the process of dialogue and discussion and promotes sharing and making sense of feelings, which is a necessary competency for IPW [17], or specifically, a process that enhances overall skills. This could be interpreted as a process that improved the care team’s ability to practice as a care team, leading to the acquisition of skills and adequate patient support.

Moreover, this study goes beyond sharing and making sense of feelings to explore essential issues and engage in dialogue and discussions about resolving them. The staff members were then able to shift their perspectives from the difficulties they experienced to the distress experienced by the patient. This was consistent with the first step of ‘understanding (knowing) the subject based on information and knowledge’ in Swanson’s theory of caring [18,19]. Moreover, the professional and ethical stance that they had to and wanted to do something about the patient’s pain facilitated the process by which all staff acquired and provided him with the new skills needed to relieve his pain, resulting in the patient regaining peace at the end of his cancer treatment. This could be interpreted as a process of caring for (being with) the patient, acting for (doing things for) the patient’s well-being, and supporting (enabling) the patient to overcome problems based on their maintained beliefs as professionals [18,19].

Swanson’s theory [18,19] suggests that the caring process leads to healing for both the patient and the practitioner. The changes in patients and practitioners in this study were considered the result of a supportive process: the healing effects brought about by the caring process.

### 4.3. Study Limitations and Challenges

This study focused on patients, and the practices listed under the categories may not be directly applicable to situations in which staff struggle to respond to frequent nurse calls or the introduction of positioning support, a new technology. However, there are currently no reports of case studies conducted using both AR and the case studies method which focus on the meaning of care. The description of how staff struggling with frequent nurse calls respond to them may be necessary in providing a solution for practitioners with similar problems. In the future, it will be essential to continue this research in the form of a practical study to gain helpful knowledge by using the case study method to explore the meaning of AR and care for practitioners who face challenges.

Moreover, this study suggests that the use of both AR and the case study method resulted in significant achievements for the nurses. While the combination of these two methodologies can be highly effective, it may also prolong the research period. Additionally, since the case study method is a relatively new research approach, there is a need for the further development of facilitators. To disseminate these practical research methods in general healthcare facilities, it is necessary to address these challenges.

## 5. Conclusions

This study demonstrated the process and practical knowledge of a practitioner struggling to support an inpatient making frequent nurse calls and gaining positive insights. Through two methodologies, AR and the case study method, nurses’ ability to support needy inpatient—careful observation, enhancement, and practical skill improvement—significantly improved. This process resulted in a better understanding of patient needs, proactive skill development, enhanced team performance, and an innovative care-delivery system that resonated throughout the ward. This study demonstrated a successful strategy for nurses to improve support for high-need inpatient, emphasising the importance of attentive care, proactive skill improvement, and a team-based approach to healthcare innovation.

## Data Availability

The data used in this study are not open to other researchers at this time.

## Supplementary Materials

The following supporting information can be downloaded at: https://www.mdpi.com/article/10.3390/nursrep14030115/s1, a comprehensive diagram summarizing figure and table.

## Public Involvement Statement

The patient was involved in this study from the implementation phase based on GRIPP2, showing genuine reactions. This led to nurses gaining deep insights. Also, in involving the patient, his privacy was protected, and informed consent was obtained in accordance with ethical guidelines.

## Guidelines and Standards Statement

This manuscript was drafted against the COREQ (Consolidated Criteria for Reporting Qualitative Research; https://www.equator-network.org/reporting-guidelines/coreq/ accessed on 10 July 2023).
