# Supplementary material for: Engaging in Action Research with Nurses: Overcoming Challenges and Gaining Positive Insights into End-of-Life Care
Source: Nurs Rep. 2024 Jun 21;14(3):1528–40. doi: 10.3390/nursrep14030115 (PMC11270203; doi:10.3390/nursrep14030115)
Supplement: Supplementary file 1 [file nursrep-14-00115-s001.zip › nursrep-2932082-supplementary.pdf]

Stage: Main actions/practices		STAGE I. Observe the patient with interest and care, grasp what the true complaint is: focus on the content of the nurse call and the patient's condition.	STAGE II. Time to act on one's own to develop the ability to provide optimal care to the patient: Participate in positioning training both inside and outside the hospital	STAGE Ⅲ. Time to raise the level of each staff member's ability to practice: Individual training on positioning is conducted and practice based on the training is started.	STAGE Ⅳ. Time to share patient changes and spread the joy of practice throughout the ward: all ward staff practice new positioning.
**Facilitator's support**		Questions that promote perspective on the patient.	Questions to facilitate reflection on positioning<br>Proposal for out-of-hospital training<br>Follow-up on out-of-hospital training: lectures and follow-up on individual	Reflection support, labor and praise for practice and results	
**Patient's situation**		He called the nurse day and night and asked them to adjust his head position, etc., and his expression of distress continued.	Continued complaints of body positional distress even after correcting posture and seeking help	Begins to spend more time calmly and without complaining of pain to the nurse	Facial expressions soften and limb contractures begin to loosen Significantly fewer complaints of pain to the nurse
1）Feeling and thinking about suffering where the subject is the patient.	(1) Why Call the Nurse? Look at the reasons why.	*①We were so bewildered by the number of nurse calls that we did not look at the background behind the patient's pressing the nurse call.*		*⑩' With positioning thus far, I now understand why the patient could not resist pressing the nurse's call!*	
	(2) Observe the patient thoroughly and assess the situation, with or without a nurse call	*②Regardless of whether the patient called for a nurse, We gathered information about the content of his complaints and facial expressions and dealt with them.*			*⑫'It can be seen that the patient's contracture is easing.*
	(3) Knowing how hard it is for the patient to position himself.	*③The complaints were mostly about position. I could tell he was in a lot of pain.*		*⑩'(Experiencing the patient's position in the training) This is painful. I thought this is not good through actual experience.*	
	(4) Accept the fact that the care was not relieving the patient's pain	*③'The number of nurse calls did not change even after adjusting the position, and this did not reduce the patient's distress.*	*④I do not know what's wrong with it. However, it cannot end like this.*		
2）Seek care tips from ourselves to improve our practice	(5) request training to PTs in the hospital to overcome the problem of positioning		*⑤Let us ask the PT so that I can position myself in a way that would suit the patient.*	*⑦The basics are important, but I was looking for a quick way to for PT.*	
	(6) Encountering care that I want to practice! I can do this!		*⑥I learned at the no-lifting training that the care I thought was good for me was actually bad for me. I thought I was doing it all the time.*		
3）Spend time on training that touches your heart	(7) Consider training methods that would move the hearts.			*⑧Training and handing out materials that convey knowledge in a light-hearted manner will not generate interest or need.*	
	(8) Steady individual follow-up until everyone realizes the difference and effectiveness of care.			*⑨If everyone feels the difference and comfort of care, it will lead to better care, and if that is the case, one-on-one practical exercises are necessary.*	
	(9) I am impressed with the comfort of the positioning! Share the feeling of "I want to give this care!			*⑩ Everyone is surprised and impressed by the difference in positioning. The atmosphere in the wards has changed as training has progress.*	
4）Create an organization that can provide new positioning care as a team	(10) Everyone can confidently provide at least one form of care based on evidence.				*⑪Not somehow, but think about putting a pillow here so that you can rest your body safely, so that you can do it with a rationale.*
	(11) Confirm the essence of the 'must-do' mindset and techniques and calling out to each other				*⑪ 'Focusing on the basics, such as supporting pillows with a stable surface rather than supporting them, so that everyone can put them into practice.*
	(12) Capture the pleasant changes that occurred in the patient and share them with all the staff.				*⑫Everyone says the contractions have loosened and blood pressure readings are easier.*

1) to 4) categories, (1) to (12) subcategories, and (1) to (12) representative codes.
